# Molecular Typing and Rapid Identification of Human Adenoviruses Associated With Respiratory Diseases Using Universal PCR and Sequencing Primers for the Three Major Capsid Genes: Penton Base, Hexon, and Fiber

**DOI:** 10.3389/fmicb.2022.911694

**Published:** 2022-05-12

**Authors:** Xiaowei Wu, Jing Zhang, Wendong Lan, Lulu Quan, Junxian Ou, Wei Zhao, Jianguo Wu, Patrick C. Y. Woo, Donald Seto, Qiwei Zhang

**Affiliations:** ^1^BSL-3 Laboratory, Guangdong Provincial Key Laboratory of Tropical Disease Research, School of Public Health, Southern Medical University, Guangzhou, China; ^2^Guangdong Provincial Key Laboratory of Virology, Institute of Medical Microbiology, Jinan University, Guangzhou, China; ^3^Foshan Institute of Medical Microbiology, Foshan, China; ^4^Department of Microbiology, The University of Hong Kong, Hong Kong, Hong Kong SAR, China; ^5^Bioinformatics and Computational Biology Program, School of Systems Biology, George Mason University, Manassas, VA, United States

**Keywords:** adenovirus, universal primers, epidemiology, molecular typing, recombination, co-infection, Hong Kong

## Abstract

Human adenoviruses (HAdVs) within species B, C, and E are responsible for highly contagious and potentially severe respiratory disease infections. The traditional method to type these pathogens was based on virus neutralization and hemagglutination assays, which are both time-consuming and difficult, particularly due to the nonavailability of reagents. Subsequent molecular typing based on the partial characterization of the hexon gene and/or the restriction enzyme analysis (REA) of the genomes is inadequate, particularly in identifying recombinants. Here, a rapid, simple, and cost-effective method for molecular typing HAdV respiratory pathogens is presented. This incorporates three pairs of universal PCR primers that target the variable regions of the three major capsid genes, i.e., hexon, penton base, and fiber genes, that span the genome. The protocol enables typing and characterization of genotypes within species B, C, and E, as well as of some genotypes within species D and F. To validate this method, we surveyed 100 children with HAdV-associated acute respiratory infections identified by direct immunofluorescence (Hong Kong; July through October, 2014). Throat swab specimens were collected and analyzed by PCR amplification and sequencing; these sequences were characterized by BLAST. HAdVs were detected in 98 out of 100 (98%) samples, distributing as follows: 74 HAdV-B3 (74%); 10 HAdV-E4 (10%); 7 HAdV-C2 (7%); 2 HAdV-C6 (2%); 1 HAdV-B7 (1%); 1 HAdV-C1 (1%); 2 co-infection (2%); and 1 novel recombinant (1%). This study is the first detailed molecular epidemiological survey of HAdVs in Hong Kong. The developed method allows for the rapid identification of HAdV respiratory pathogens, including recombinants, and bypasses the need for whole genome sequencing for real-time surveillance of circulating adenovirus strains in outbreaks and populations by clinical virologists, public health officials, and epidemiologists.

## Introduction

Human adenoviruses (HAdVs) belong to the *Adenoviridae* family, which are nonenveloped double-stranded DNA viruses (Lion, 2014). As human pathogens, HAdVs are responsible for a wide spectrum of diseases in the respiratory, ocular, gastrointestinal, and renal tracts, commonly (Lion, 2014). A measure of its importance as a respiratory pathogen, for example, and its pathogenicity attributes includes being attributed to approximately 5%–7% of the respiratory illnesses diagnosed in young children in four cities of Argentina during 1993–1994 (Carballal et al., 2001). These respiratory pathogens are highly contagious and can spread rapidly in crowded places such as hospitals, schools, the military, and newborn nurseries (Lynch and Kajon, 2016). Although respiratory tract infections (RTIs) caused by HAdVs are generally self-limiting and may even be mild, a number of severe and fatal infections have been reported in both children and adults (Carballal et al., 2001; Rebelo-de-Andrade et al., 2010; Carr et al., 2011; Cui et al., 2015; Yu et al., 2016; Zhang S. et al., 2016).

To date, 113 HAdV genotypes have been identified, characterized, and reported using whole genome analysis, including the original 51 serotypes^1^; these are parsed into species A–G, partially based on biological and pathogenicity attributes (Lion, 2014). Among these seven species, species B types (HAdV-B3, -B7, -B14, -B16, -B21, and -B55) and one species E type (HAdV-E4) are commonly associated with acute respiratory disease (ARD), which accounts for a high proportion of respiratory diseases in both children and adults (Madisch et al., 2006; Zhang et al., 2006, 2012b; Kajon et al., 2007; Rebelo-de-Andrade et al., 2010; Girouard et al., 2011; Zhao et al., 2014; Chen et al., 2016; Zeng et al., 2016; Cheng et al., 2018; Jing et al., 2019). The types in species C (HAdV-C1, -C2, -C5, -C6, and -C57) are generally associated with mild respiratory diseases and latent infections, but are important in immunocompromised patients (Lion, 2014). HAdVs comprising species D cause ocular and gastrointestinal diseases. Species A, F, and G types are associated with gastroenteritis (Lu et al., 2014). The predominant types are different among different countries or regions and change over time (Erdman et al., 2002; Chang et al., 2008; Lee et al., 2010). HAdV types C1 to B7 account for more than 80% of the HAdV infections in infants and children (Piedra et al., 1998). Globally, HAdV-B3 is among the most common types implicated in HAdV respiratory infections in children and adults (Schmitz et al., 1983; Gray et al., 2007; Chang et al., 2008; Yan et al., 2021). In South America, HAdV-B7 has been a predominant strain associated with RTIs requiring hospitalization in many countries (Li et al., 1996; Carballal et al., 2001). In Asia, HAdV-B3 and -B7 have been the predominant types associated with RTIs in children (Li et al., 1996; Lin et al., 2004; Chang et al., 2008; Tsou et al., 2012; Han et al., 2013; Zhao et al., 2014; Chen et al., 2016; Wang et al., 2016; Yu et al., 2016; Yan et al., 2021), while HAdV-B7 are associated with higher severity of illness and fatality rate (Zhao et al., 2014; Chen et al., 2016; Yu et al., 2016). In Europe, HAdV-B3 and HAdV-B7 are highly virulent and potentially deadly types, especially for children (Lion, 2014). Historically, types HAdV-B7 and -E4 predominate as a cause of ARD among military personnel in the United States (Sanchez et al., 2001; Erdman et al., 2002; Kolavic-Gray et al., 2002; Kajon et al., 2007).

In immunocompromised patients, particularly in the organ transplant setting, the most commonly reported adenovirus types include HAdV-C1, -C2, -C5, -A12, -A31, -B3, -B11, -B16, -B34, and -B35 (Barrero et al., 2012). These types may be associated with higher and more severe morbidity and mortality outcomes (Lion, 2014).

As a result, it is important to type HAdVs accurately and rapidly for clinical diagnoses and epidemiological investigations in order to provide information on the pathogen, including the distribution of infections by individual and specific types, as well as to detect and characterize emergent strains in the context of outbreaks (Xie et al., 2013; Li et al., 2014; Dongliu et al., 2016; Tan et al., 2016; Yi et al., 2017). As recombination is recognized as a significant evolutionary pathway for the emergence of novel HAdV pathogens, rapid characterization is important (Walsh et al., 2009, 2010; Zhou et al., 2012; Dehghan et al., 2013a, 2019; Robinson et al., 2013a,b).

HAdVs were traditionally typed according to serum neutralization and hemagglutination-inhibition tests, which are time-consuming and reagent-limited (Wigand, 1987; Seto et al., 2013).

Molecular typing based on either partial sequence characterization of HAdV hexon gene or restriction enzyme analysis (REA) of the genomics DNA are improvements but still not effective, for example, in their difficulty to identify recombinants (Seto et al., 2013). HAdV isolates with identical serum-neutralizing attribution but with unexpected biological or pathogenic characteristics have been reported (Walsh et al., 2010; Kaneko et al., 2011; Matsushima et al., 2011; Zhou et al., 2012), challenging the traditional view of “hexon-centric” identification. With the recent development in whole genome sequencing and bioinformatics analysis, a wider range of HAdV genomes from clinical isolates have been sequenced and analyzed (Seto et al., 2010). An important finding is that recombination, scored by characterizing the three major capsid genes, i.e., penton base, hexon, and fiber genes, contributes substantially to the genesis of novel and emergent pathogenic HAdVs. Among the 61 recent novel pathogenic genotypes identified and recognized since HAdV-52, nearly all are recombinants (Ishiko et al., 2008; Ishiko and Aoki, 2009; Walsh et al., 2009, 2010, 2011; Liu et al., 2011, 2012; Matsushima et al., 2011; Robinson et al., 2011; Zhang et al., 2012a; Hage et al., 2015; Cheng et al., 2018). A striking example is HAdV-B55. This is a “Trojan horse,” as it is a highly contagious human respiratory pathogen that is a recombinant of HAdV-B11 and HAdV-B14 parentals (Yang et al., 2009; Zhang et al., 2012a; Dongliu et al., 2016; Cheng et al., 2018). It has a HAdV-14 genome chassis, including the HAdV-14 penton base gene and fiber gene, which encodes cell tropism (Pan et al., 2018), but a partial HAdV-11 hexon gene, which encodes the antigenic epitopes of the virus denoting it as a renal pathogen. This virus possesses the biological and pathogenic attributes of HAdV-14 and also avoids the neutralizing antibody against HAdV-14 in a population. It was noted to be previously mistyped as HAdV-11a by partial hexon sequencing and REA due to incomplete gene analysis and incorrect application of the REA method (Walsh et al., 2010; Seto et al., 2013; Zhang Q. et al., 2016).

Whole genome sequencing is very useful for epidemiological surveys and understanding archived intriguing pathogens (Ismail et al., 2018; Dehghan et al., 2019; Coleman et al., 2020) but may still be unfeasible for the routine large-scale molecular epidemiological monitoring of nonpandemic outbreaks, as well as for the rapid identification of viral pathogens during outbreaks (Zhu et al., 2009; Xie et al., 2013). Therefore, to circumvent the limitations of using only the hexon for sampling adenoviral pathogens and also to ascertain the high sequence diversity between different HAdV species, we developed a simple, rapid, cost-effective, practical, and universal typing method for the routine epidemiological surveillance of human respiratory adenoviruses.

At the same time, we characterized the molecular epidemiology of HAdVs circulating among inpatient and outpatient children during the ARD outbreaks in the late summer and early autumn of 2014 in Hong Kong, using our newly-developed HAdV molecular typing protocol. This is the first detailed molecular epidemiological survey of HAdVs circulating in Hong Kong.

## Materials and Methods

### Viruses and Other Materials

Human adenovirus genotypes HAdV-B3, -B7, -B11, -B14, -B21, -B55, -C5, -D19, -E4, and -F41 have been isolated, identified, studied, and archived in our laboratory (Zhang et al., 2006, 2012a,b, 2017; Zhao et al., 2014; Yu et al., 2016). Molecular analysis have entailed the use of Taq PCR Master Mix kits (Takara Corp.; Japan), QIAamp DNA Mini kits (QIAgen Corp.; China), and PCR cleanup kits (Axygen Inc.; United States), applied according to the manufacturers’ instructions. For characterization and reference, DL10000 and DL2000 DNA Markers were used, and are the products of Takara (Takara Corp.; Japan).

### Clinical Specimens

This is a retrospective survey of an ARD outbreak in pediatric outpatients and inpatients with influenza-like symptoms in Queen Mary Hospital, Hong Kong from July 2014 through October 2014. For further details on the samples, please see Zhang et al. (2019). Briefly, nasopharyngeal swab specimens were collected and adenoviruses were detected using a Direct Immunofluorescence IMAGEN™ Adenovirus Detection Kit (Thermo Fisher; United States). Specific genotypes were identified and presented in this report.

The study protocol was approved by the Institutional Ethics Committee of Queen Mary Hospital in accordance with its guidelines for the protection of individual privacy, and adhering to the principles of the Declaration of Helsinki. Patient consent for using left-over specimens was waived.

### Adenovirus Culturing and Isolation

The HAdV-positive nasopharyngeal swab specimens collected from 100 patients were inoculated onto A549 cells. These were cultured in a maintenance medium (Minimal Essential Medium containing 2% fetal bovine serum, 100 U/ml penicillin G, and 100 μg/ml streptomycin) at 37°C in an atmosphere containing 5% (v/v) carbon dioxide. Cytopathic effect (CPE) was monitored for 5–7 days, following which, if no CPE was observed, the cells would be frozen and thawed three times and then passaged in A549 cells again to check for CPE.

### PCR Primers Design and PCR Amplification

The penton base, hexon, and fiber gene sequences from HAdV-A18, -B3, -B7, -B11, -B14, -B16, -B21, -B34, -B35, -B50, -B55, -B66, -B68, -C1, -C2, -C5, -C6, -D19, -D37, -E4, -F41, and -G52 were extracted from genome data archived in GenBank. These were aligned using ClustalW to find regions with high sequence similarities. Primers targeting the three major capsid genes were designed based on these bracketing conserved regions, and these were subsequently obtained from Invitrogen (Guangzhou, China).

PCR conditions were determined, and reactions were conducted in a total volume of 20 μl comprising 1× Taq Master Mix (10 μl), primer F (10 μmol/L, 0.5 μl), primer R (10 μmol/L, 0.5 μl), DNA template (1 μl), and water (8 μl). Primers Penton-F and Penton-R were designed for both the PCR amplification and the subsequent DNA sequencing of the amplified penton base gene product. PCR conditions are as follows: 94°C for 1 min; 34 cycles of 94°C for 30 s, 52°C for 30 s, and 72°C for 100 s; and a final extension of 72°C for 10 min. Similarly, Primers HVR-F and HVR-R were used for both the PCR amplification and subsequent DNA sequencing of the hexon gene product. Hexon PCR conditions are as follows: 94°C for 1 min; 34 cycles of 94°C for 30 s, 52°C for 30 s, and 72°C for 100 s; followed by a final extension of 72°C for 10 min. Finally, Primers Fiber-F and Fiber-R were designed for both the PCR amplification and subsequent DNA sequencing of the fiber gene product. The PCR amplification conditions for this are as follows: 94°C for 1 min; 34 cycles of 94°C for 30 s, 52°C for 30 s, and 72°C for 72 s; followed by a final extension of 72°C for 10 min.

### Sequencing and Molecular Typing of Clinical Adenovirus Specimens

All three major capsid genes of the HAdV isolates were PCR-amplified using the corresponding pairs of universal primers noted above, respectively. These PCR products were purified and then DNA-sequenced directly with both PCR primers. The assembled DNA sequences were characterized by a BLAST survey of the NCBI GenBank database. Genotype identity of each clinical specimen was determined based on the penton base, hexon, and fiber sequences to which they showed the highest sequence identities.

### Genome Reference Sequences Used for Alignments and Primer Design

As noted, archived genome sequences from GenBank were used for the alignments and extraction of the penton base, hexon, and fiber gene sequences. Their accession numbers are as follows: HAdV-A12 (X73487), HAdV-B3 (DQ099432), HAdV-B3 (AY599834), HAdV-B7 (AY594255), HAdV-B7 (KC440171), HAdV-B11 (AY163756), HAdV-B14 (AY803294), HAdV-B16 (AY601636), HAdV-B21 (AY601633), HAdV-B34 (AY737797), HAdV-B35 (AY128640), HAdV-B50 (AY737798), HAdV-B55 (JX491639), HAdV-C1 (AC_000017), HAdV-C2 (J01917), HAdV-C5 (AC_000008), HAdV-C6 (KF268129), HAdV-D9 (AJ854486), HAdV-E4 (AY594253), HAdV-F40 (KU162869), and HAdV-G52 (DQ923122).

### Phylogenetic Analysis

Molecular Evolutionary Genetics Analysis (MEGA) software, version 7.0,^2^ a was used for the phylogenetic analyses of the penton base, hexon, and fiber genes as determined from the clinical specimens, along with additional sequences, for references, that were retrieved from GenBank. Phylogenetic trees were constructed using the maximum parsimony method with a bootstrap test of 1,000 replicates and the Tree-Bisection-Reconnection (TBR) model.

## Results

### Three Pairs of Universal Primers Amplify the Three Major Capsid Genes: Penton Base, Hexon, and Fiber Genes

**Figure 1:**
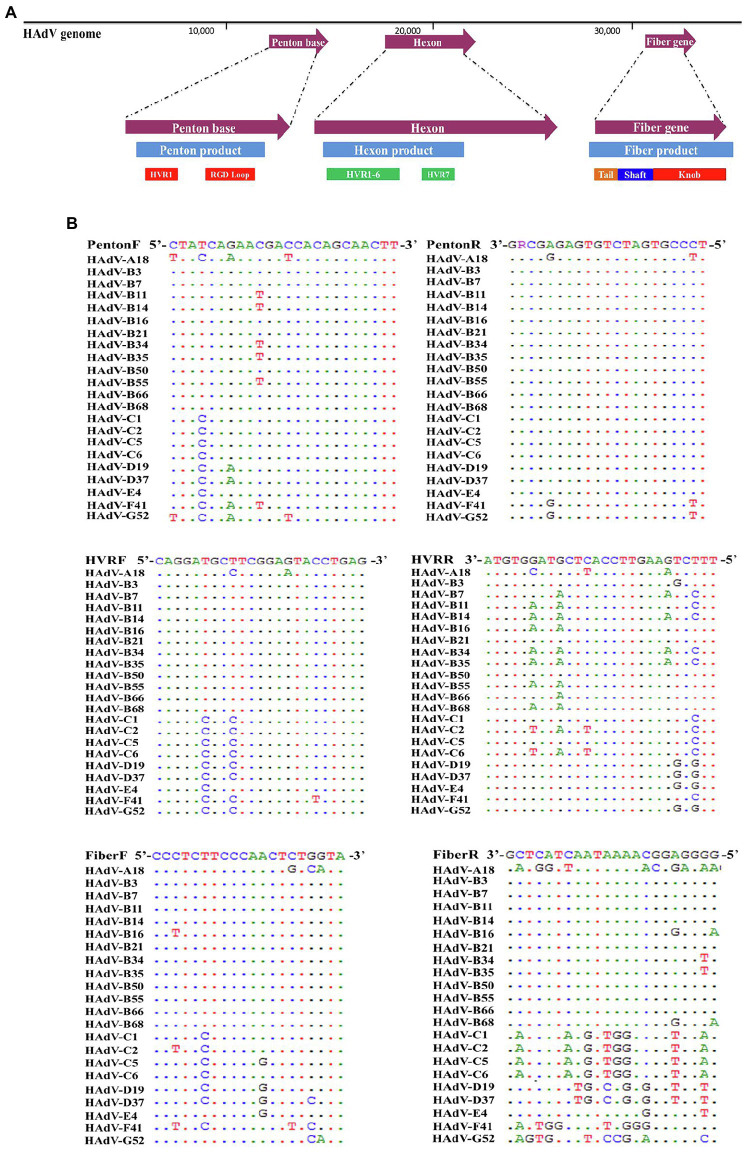
**(A)** Schematic human adenoviruses (HAdV) genome, with the primer positions and the resultant PCR products noted for the three major capsid protein genes: penton base (left), hexon (middle), and fiber (right) genes. The relative locations of the capsid protein genes in the HAdV genome are also noted. These genes contain the genotyping and molecular characterization information for HAdVs. The purple arrows indicate the genes and their locations; the blue bars indicate the PCR products and their relative lengths; and other colored bars indicate important domains within each gene, including hypervariable regions (HVRs and RGD Loop), as well as the tail, shaft, and knob domains. **(B)** Alignment of HAdV universal primers for HAdV-A18, B3, -B7, B-11, -B14, -B16, -B21, -B34, -B35, -B50, -B55, -B66, -B68, -C1, -C2 -C5, -C6, -D9, -D19, -D37, -E4, -F41, and -G52. Divergence from the primer sequences is shown for the isolates tested. Each virus is identified with its genotype number as well as its species demarcation for reference. Dots represent identical bases, and base differences are noted. The bases are color-coded for visual comparisons.

Three pairs of universal primers were designed for PCR amplification and DNA sequencing of the HAdV penton base, hexon, and fiber genes. Primers Penton-F and Penton-R were designed based on the conserved regions of the penton base sequences. Within the alignment of sequences, primer sequences selected for Penton-F and Penton-R are highly conserved in the majority of penton base genes, which ensures that all of the analyzed adenovirus types within species A to G could be PCR-identified (Figure 1B). The resultant PCR product is 1,253 bp (Table 1), located within penton base gene and encompasses the variable regions HVR1 and RGD loop (Figure 1A).

**Table 1:** Universal primers for the detection, typing, and sequencing of HAdV.

Gene	Position	Length^a^ (bp)	Primer	Primer sequence	Position^a^	PCR product	PCR condition
Penton base	13,904–15,538	1,635	Penton-F	5’-CTATCAGAACGACCACAGCAACTT-3’	14,152–14,175	1,253 bp	34 cycles of 94°C for 30 s; 52°C for 30 s; and 72°C for 100 s
			Penton-R	5’-TCCCGTGATCTGTGAGAGCRG-3’	15,384–15,404		
Hexon	18,422–21,256	2,835	HVR-F	5’-CAGGATGCTTCGGAGTACCTGAG-3’	18,473–18,495	1,685 bp	
			HVR-R	5’-TTTCTGAAGTTCCACTCGTAGGTGTA-3’	20,132–20,157		
Fiber	31,301–32,260	960 (1278^b^)	Fiber-F	5’-CCCTCTTCCCAACTCTGGTA-3’	31,180–31,199	1,153 bp (1,519 bp^b^)	
			Fiber-R	5’-GGGGAGGCAAAATAACTACTCG-3’	32,311–32,332		
		1746^c^	Fiber-CR	5’-GAGGTGGCAGGTTGAATACTAG-3’	32,311–32,332	2027 bp^c^	

*Sequences, genome locations, and the resultant predicted PCR product sizes are noted for each of the three major capsid protein genes. Fiber-CR is the species C-specific optimal primer due to their divergence from other HAdVs*.

a *Positions are in the reference genome of HAdV-B3 (GenBank acc. no. DQ099432)*.

b *The length of HAdV-4 fiber gene, for reference*.

c *The length of the PCR product of HAdV-C fiber gene, for reference*.

For the hexon, the universal amplification primers HVR-F and HVR-R were designed similarly. The primer sequences are conserved in HAdV genotypes across all of the species analyzed, yielding a PCR product of about 1,685 bp (Table 1). This amplicon contains the seven hypervariable regions (HVRs) comprising Loops 1 and 2 (Figure 1A), which are the epitope determinants used for serotyping.

Primers Fiber-F and Fiber-R were designed for fiber gene. Point mutations of primer Fiber-F exist only in one or two nucleotides located in the middle of the primers; this ensures an effective PCR amplification of the different HAdV genotypes (Figure 1A). These primers amplify HAdV genotypes from species B, D, and E, yielding a PCR product of about 1,153 bp for HAdV-B and -D, and 1,519 bp for HAdV-E4. However, due to higher sequence variation and the longer fiber gene (about 1746 bp) of the species C HAdVs, Fiber-R matches poorly with these genotypes in HAdV-C. To compensate, another primer Fiber-CR was designed to match completely the sequences within species C (Table 1). This provides a product that is 2027 bp.

### Universal Primers-Based PCR Amplification and Sequencing Provides Identification of Reference HAdV-B3, -B7, -B11, -B14, -B21, -B55, -C5, -D19, -E4, and -F41

**Figure 2:**
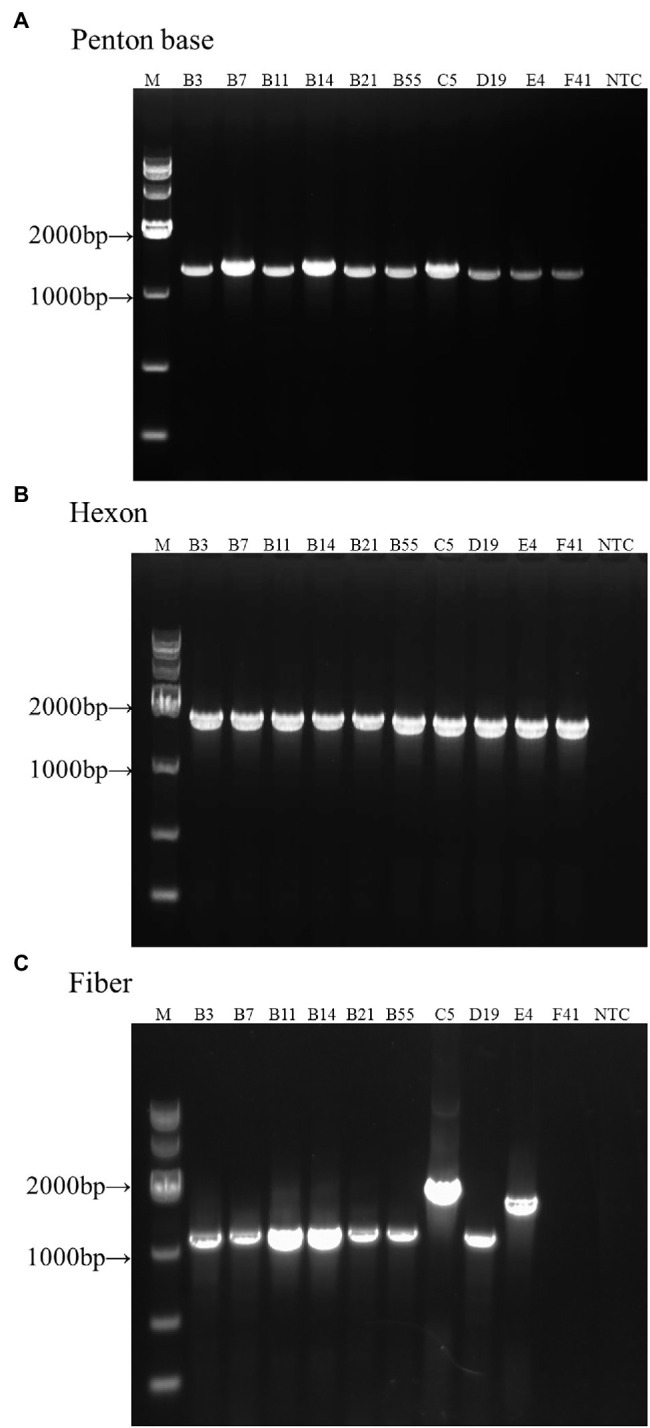
One percent Agarose gel electrophoresis profiles of PCR products from HAdV-B3, -B7, -B11, -B14, -B21, -B55, -C5, -D19, -E4, and -F41 using universal primers for penton base, hexon, and fiber genes. **(A)** Penton base gene PCR amplification using primers Penton-F and Penton-R. **(B)** Hexon gene PCR amplification using primers HVR-F and HVR-R. **(C)** Fiber gene PCR amplification using primers Fiber-F and Fiber-R (Fiber-CR for HAdV-C5). Molecular weight sizing markers are in lane M. NTC, “no template” PCR control.

Genomic DNA from reference samples of HAdV-B3, -B7, -B11, -B14, -B21, -B55, -C5, -D19, -E4, and -F41 was extracted and amplified by PCR amplification and identified by subsequent sequencing using these three pairs of universal primers (Figure 2). The PCR products were specific, yielding single distinct products, the expected predicted sizes: 1.2 kb (penton base), 1.6 kb (hexon), and 1.1 kb (fiber), respectively. However, there are two exceptions: one is the HAdV-E4 fiber gene (1,519 bp) and the other is the HAdV-C5 fiber gene (2027 bp). Both gene products are longer than their counterparts in the other HAdV genotypes. The species C fiber requires an alternative primer, Fiber-CR.

### The Epidemiological Survey of HAdVs in Hong Kong From 100 Pediatric ARD Patients in 2014

A total of 100 HAdV-positive samples were analyzed using this protocol (Table 2). These indicated that the male and female rates were 50% and 49%, respectively (one was of unrecorded gender). Therefore, no significant gender difference was found. The median age with HAdV infection was 4 years, ranging from 0.5 to 14 years old, of which 60% were under 5 years. Of the 100 cases, 93 (93%) were hospitalized, with the median hospitalization duration of 3 days (1–36 days). Fourteen HAdV-positive cases were also co-infected with EV/RV; two co-infected with RSV, and one co-infected with influenza C.

**Table 2:** Comparison of demographic and clinical characteristics of 100 children with ARD according to HAdV type in Hong Kong, summer 2014.

HAdV species		All	HAdV-B3	HAdV-C1	HAdV-C2	HAdV-E4	HAdV-C6	HAdV-B7	Recombinati-on or co-infection	Undetectable
Numbers		100	74	1	7	10	2	1	3	2
Gender (M/F)		50/49	38/35	0/1	5/2	3/7	1/1	1/0	2/1	0/2
Age (range)		4 (0.5–14)	2.5 (1–14)	4 (4)	3 (0.5–7)	5 (1–11)	3.5 (3–4)	7 (7)	2 (1–9)	3.5 (3–4)
Nationality	Chinese	86	63	1	5	9	2	1	3	2
	Occidental	2	2	-	-	-	-	-	-	-
	Korean	1	1	-	-	-	-	-	-	-
	Indian	1	1	-	-	-	-	-	-	-
	Nepalese	3	1	-	2	-	-	-	-	-
Hospitalized numbers		93	68	1	7	9	2	1	3	2
Hospitalized day (range)		3 (1–36)	3 (1–36)	3 (3)	3 (2–4)	2 (1–15)	2.5 (2–3)	4 (4)	2 (2–3)	3 (3)
Co-infection	EV/RV	14	10	-	1	2	-	-	-	1
	RSV	2	1	-	-	1	-	-	-	-
	Flu C	1	1	-	-	-	-	-	-	-
Clinical diagnosis										
URTI		74	55		6	8	1		2	1
Diarrhea		5	3	1					1	
Bronchiolitis		3	2							1
Febrile seizure		3	3							
Pneumonia^*^		1	1^*^							
Rash		2	2							
Adenoviremia transplant recipient		1	-			1				
Conjunctivitis/Febrile seizure		1	1							
Fever/GE		1	-		1			1		
Intussusception		1	-				1			
N/A		8	7			1				

* *Death*.

The clinical diagnoses included one case of fatal pneumonia caused by HAdV-3; three cases of bronchitis; three cases of diarrhea; two cases of febrile seizure; two cases of rash; and 74 cases of upper respiratory tract infection (URTI; Table 2).

### Molecular Typing of HAdV Clinical Samples

**Figure 3:**
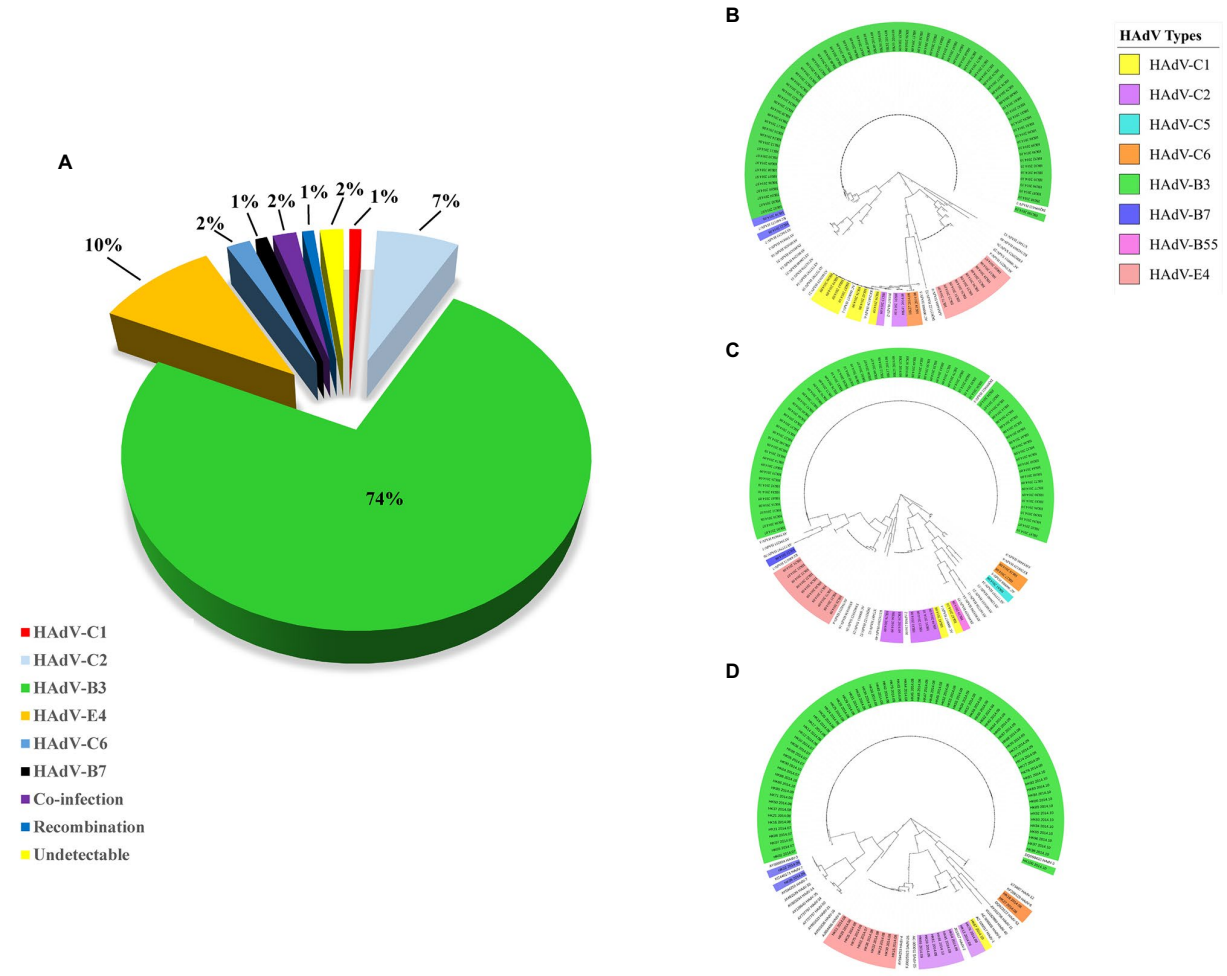
**(A)** HAdV genotype distribution in hospitalized pediatric patients with ARIs from July through October, 2014. Panels **(B–D)** display the phylogenetic relationships of the penton base, hexon, and fiber genes, respectively, of the 98 identified HAdV clinical isolates. Additional reference sequences were retrieved from GenBank to provide context and reference. The phylogenetic trees were generated based on the Tree-Bisection-Reconnection (TBR) model by MEGA 7.0 (www.megasoftware.net/) using the maximum parsimony method with 1,000 boot-strap replicates and default parameters. The percentage of trees in which the associated taxa clustered together is shown next to the branches. The scale bar is in units of nucleotide substitutions per site.

Hundred throat swabs specimens were successfully amplified by this PCR protocol using the three pairs of universal primers. These PCR products were sequenced using the same primers. BLAST analysis confirmed that 74 cases were HAdV-B3 (74%); 10 cases were HAdV-E4 (10%); seven cases were HAdV-C2 (7%); two cases were HAdV-C6 (2%); one case was HAdV-B7 (1%); one case was HAdV-C1 (1%); two cases were co-infected by different types of HAdVs (HAdV-B7 and HAdV-B55; HAdV-C1 and HAdV-B3) (2%); one case was putative recombinant (P1H2F2) (1%); the remaining two cases were PCR negative (Figure 3A). The two cases co-infected by different HAdV types were identified by double peaks emergent in the sequencing maps, i.e., either set of stripped peaks were identical with HAdV-B7 and HAdV-B55, respectively, or identical with HAdV-C1 and HAdV-B3. One case identified as putative recombinant showed clear and single-peaked for each base.

### Phylogenetic Analysis of HAdV Clinical Samples

Phylogenetic analysis of the hexon, penton base, and fiber gene of the Hong Kong clinical samples is shown in Figure 3. The phylogenetic analysis results are consistent with the BLAST results. Panels (B), (C), and (D) display the phylogenetic relationships of the penton base, hexon, and fiber genes, respectively. Of these 100 clinical HAdV isolates, HAdV-B3 (*n* = 74) was the most prevalent type. The three capsid genes formed a subclade with another China HAdV-3 isolate Guangzhou01 circulating in 2004 (Zhang et al., 2006). HAdV-E4 was the second most prevalent strain (*n* = 10). This is unexpected as HAdV-E4 was formerly and apparently constrained to military populations (van der Veen et al., 1969; Kolavic-Gray et al., 2002). However, this may not be surprising as a recombination of a viral replication motif, NF-I, that may have resulted in host adaption and allowed HAdV-E4 to infect a wider population was reported (Dehghan et al., 2013b; Zhang et al., 2019; Coleman et al., 2020).

### GenBank Accession Numbers of the Capsid Protein Genes Sequenced From the Clinical Isolates

PCR products derived from the penton base, hexon, and fiber genes of the throat swab specimens were sequenced. Of these, sequences from each representative type were submitted to GenBank, and their GenBank accession numbers are summarized in Table 3.

**Table 3:** Genotyping and GenBank accession numbers of the clinical isolates.

Strain no.	Gene	Adenovirus Genotype	GenBank accession no.
HK18	Penton base	E6	OM988092
	Hexon		OM988093
	Fiber		OM988091
HK22	Penton base	B7	OM988085
	Hexon		OM988086
	Fiber		OM988084
HK35	Penton base	E4	QVS02965.1
	Hexon		QVS02970.1
	Fiber		QVS02985.1
HK39	Penton base	B7	OM988087
	Hexon	B55(double peaks)	OM988088
	Fiber	B7	OM988083
HK42	Penton base	C1	OM988098
	Hexon	C1	OM988097
	Fiber	B3 and C1(double peaks)	OM988094, ON098955
HK61		P1H2F2	ON054624
HK76	Penton	C2	OM988100
	Hexon		OM988096
	Fiber		OM988095
HK87	Penton	C1	OM988099
	Hexon		OM988090
	Fiber		OM988089
HK91	Penton	C2	AVZ45764.1
	Hexon		AVZ45769.1
	Fiber		AVZ45784.1

## Discussion

PCR and DNA sequencing analysis of microbial DNA has been used for quick identification and better characterization of the pathogens (Tang et al., 1997). In the past, the identification of HAdV genotypes was traditionally performed by hexon protein analysis, e.g., virus neutralization, which depended on the interaction between serotype-specific antisera and serotype-specific antigenic epitopes in HVRs of the hexon protein (Dudding et al., 1972), or, later, by limited hexon gene sequencing (Lu and Erdman, 2006). The time-consuming and labor-intensive epitope detection method is seldom used today; the limited partial hexon sequencing provides partial and incomplete identification, as any recombinant HAdV will be missed. As a gold standard, whole genome sequencing and analysis is the most accurate method to identify, characterize, and type HAdVs properly. This is borne out by recent recognition of new genotypes that include recombinant HAdVs identified by this whole genome analysis method, including important pathogenic and emergent HAdVs. As an example, HAdV-D53 was recognized as a new genotype because genome recombination was detected amongst the three major capsid genes that span the genome. The penton base, hexon, and fiber genes originated from genotypes HAdV-D37, HAdV-D22, and HAdV-D8, respectively (Walsh et al., 2009). While HAdV-D22 is nonpathogenic, HAdV-D53 is a potent and highly contagious epidemic keratoconjunctivitis ocular pathogen (Walsh et al., 2009). Another example is the emergent genotype HAdV-B55 (Zhu et al., 2009; Walsh et al., 2010). This is a highly contagious respiratory pathogen that is a recombinant containing the hexon epitope from a urinary tract pathogen HAdV-B11 along with the penton base and fiber genes from a respiratory tract pathogen HAdV-B14.

Given that whole genome sequencing is still relatively cost-prohibitive, particularly for large numbers of samples comprising outbreak and population sampling projects, and that genome recombination may only be indicated by assaying marker genes across the genome, such as the penton base, hexon, and fiber genes, a simple, rapid, cost-effective, practical, and universal detection and typing method for characterizing HAdVs is presented in this study. This protocol calls for using three pairs of universal PCR primers to target variable regions of the three capsid genes to provide products for characterizing the adenoviral isolates. The subsequent amplicon sequencing and BLAST analysis provides information as to the genotype identity and also whether there is any recombination across the genome. This method was validated by typing 98 clinical specimens successfully. In practice, all three pairs of universal primers that were chosen and optimized have worked for the genotyping of HAdV-B3, -B7, -B11, -B14, -B21, -B55, -C5, -D19, and -E4. However, because the HAdV-C fiber gene sequences are phylogenetically distinct from the other species, to compensate, a specific primer Fiber-CR was designed. This worked well for the HAdV-C fiber amplification. The universal primers for penton base and hexon genes successfully amplified the genotypes from across species B to F, even though the genomic sequences between different species are diverse. Isolates from the set of genotypes representing species HAdV-B, -C, -D, and -E can be detected and type-identified by this protocol using these universal primers. These include putative recombinants, for example, the HAdV-B55 isolate.

In contrast to a commonly used HAdV typing protocol published by Lu and Erdman (2006) and others which were based solely on the PCR amplification and/or sequencing of the HAdV hexon gene (Pring-Akerblom et al., 1999; Takeuchi et al., 1999; Sarantis et al., 2004; Zhu et al., 2009), the method presented in this report is more informative. Additionally, the Lu and Erdman (2006) protocol targets only the HVR1-6 rather than the entire epitope. Although HVR1-6 do contain type-specific epitopes, the adjacent HVR-7 region does as well (Sarantis et al., 2004; Yuan et al., 2009). As noted, these hexon-centric methods do not identify recombination across the genome, which appears to be an important molecular evolution mechanism in the genesis of novel and emergent HAdVs, as noted by the recent characterization and recognition of several emergent human adenoviral pathogens (Ishiko et al., 2008; Ishiko and Aoki, 2009; Walsh et al., 2009, 2010, 2011; Liu et al., 2011, 2012; Matsushima et al., 2011; Robinson et al., 2011; Zhang et al., 2012a; Hage et al., 2015). McCarthy, et al. proposed a clinical algorithm for detecting HAdV coinfections and strains by PCR amplification and sequencing of subregions of the hexon and fiber genes (McCarthy et al., 2009). However, the strains with recombination including the penton base gene, and the 5’-end of the genome, would be missed. For example, HAdV-D53 was a recombinant strain with a penton base of HAdV-37, hexon of HAdV-22, and fiber gene of HAdV-8 (Walsh et al., 2009). Another example is HAdV-D86, which contained hexon and fiber genes of HAdV-25 but penton base gene of HAdV-9 (P9H25F25; GenBank accession number KX868297; Kajan et al., 2018). On the contrary, our PCR amplification and DNA sequencing method not only targets three genes that essentially span the genome, but also includes the seven HVRs, which will identify any recombination within the hexon epitopes as well. The three PCR reactions can be performed concurrently and rapidly, optimizing detection time. This method will economically provide the identification and characterization of HAdVs, particularly recombinants, in the real-time surveillance, sampling, and screening of circulating large numbers of HAdV isolates during outbreaks and in populations for clinical microbiologists, public health officers, and epidemiologists.

## Data Availability Statement

The datasets presented in this study can be found in online repositories. The names of the repository/repositories and accession number(s) can be found in the article/supplementary material.

## Ethics Statement

The studies involving human participants were reviewed and approved by Institutional Ethics Committee of Queen Mary Hospital. Written informed consent for participation was not required for this study in accordance with the national legislation and the institutional requirements.

## Author Contributions

DS and QZ contributed to the study design and manuscript writing. XW, JZ, WL, LQ, JO, PW, and QZ contributed to data analysis and data visualization. WZ, JW, DS, and QZ contributed to manuscript revision. All authors contributed to the article and approved the submitted version.

## Funding

This work was supported by grants from the National Key Research and Development Program of China (2018YFE0204503), the National Natural Science Foundation of China (32170139 and 81730061), the Natural Science Foundation of Guangdong Province (2018B030312010, 2021A1515010788, and 2022A1515011190), and the Fundamental Research Funds for the Central Universities (21622101).

## Conflict of Interest

The authors declare that the research was conducted in the absence of any commercial or financial relationships that could be construed as a potential conflict of interest.

## Publisher’s Note

All claims expressed in this article are solely those of the authors and do not necessarily represent those of their affiliated organizations, or those of the publisher, the editors and the reviewers. Any product that may be evaluated in this article, or claim that may be made by its manufacturer, is not guaranteed or endorsed by the publisher.

## Footnotes

^1^ http://hadvwg.gmu.edu/

^2^ https://www.megasoftware.net/
